# Research Progress in Pharmacological Activities and Applications of Cardiotonic Steroids

**DOI:** 10.3389/fphar.2022.902459

**Published:** 2022-06-02

**Authors:** Junwei Ren, Xinyuan Gao, Xi Guo, Ning Wang, Xin Wang

**Affiliations:** ^1^ Key Laboratory of Cardiovascular Medicine Research, Department of Pharmacology, Ministry of Education, Harbin Medical University, Harbin, China; ^2^ Thyroid Surgery, Affiliated Cancer Hospital, Harbin Medical University, Harbin, China; ^3^ Department of Pharmacy, The Fourth Affiliated Hospital of Harbin Medical University, Harbin, China

**Keywords:** cardiotonic steroids (CTS), antitumor, antiviral, neuroprotection, immune regulation

## Abstract

Cardiotonic steroids (CTS) are a group of compounds existing in animals and plants. CTS are commonly referred to cardiac glycosides (CGs) which are composed of sugar residues, unsaturated lactone rings and steroid cores. Their traditional mechanism of action is to inhibit sodium-potassium ATPase to strengthen the heart and regulate heart rate, so it is currently widely used in the treatment of cardiovascular diseases such as heart failure and tachyarrhythmia. It is worth noticing that recent studies have found an avalanche of inestimable values of CTS applications in many fields such as anti-tumor, anti-virus, neuroprotection, and immune regulation through multi-molecular mechanisms. Thus, the pharmacological activities and applications of CTS have extensive prospects, which would provide a direction for new drug research and development. Here, we review the potential applications of CTS in cardiovascular system and other systems. We also provide suggestions for new clinical practical strategies of CTS, for many diseases. Four main themes will be discussed, in relation to the impact of CTS, on 1) tumors, 2) viral infections, 3) nervous system diseases and 4) immune-inflammation-related diseases.

## 1 Introduction

CTS are a class of organic compounds with a long history of applications and are now widely used to treat heart failure. Recently, at home and abroad scholars have carried out extensive and intensive research on CTS and found them have various pharmacological activities (Wang et al., 2001; Ayogu and Odoh, 2020; Škubník et al., 2021b; Jansson et al., 2021). They are expected to be used more widely in the medical field. CTS exist in plants and can also be synthesized by the human body. Therefore, they can be classified as endogenous or exogenous by source. Exogenous mainly comes from Scrophulariaceae and Apocynaceae plants, such as Digitalis lanata, Nerium oleander, Digitalis purpurea and so on (Deng et al., 2020). Currently, the clinical preparations include digitoxin, digoxin, lantoside C and strophanthin K. Endogenous CTS are endogenous digitalis-like factors (EDLF) found in mammalian tissues and body fluids (including the brain, adrenal gland, plasma, urine, and placenta, etc.), such as ouabain-like substances found in human plasma and adrenal glands, digitalis-like substances found in human urine and placenta, ouabain-like substances extracted from bovine hypothalamus (Goto et al., 1990; Hamlyn et al., 1991; Tymiak et al., 1993; Doris et al., 1996; Ma et al., 2012). Whether they are endogenous or exogenous, the basic structure of CTS determines that they have similar effects, and different modification groups provide them with rich pharmacological activities. The mechanism by which CTS bind to and inhibit the Na^+^-K^+^-ATPase (NKA, sodium and potassium pump) is widely accepted. In non-traditional mechanisms, NKA still plays a predominant role. For instance, ouabain inhibit NKA and lead to a progressive decline in membrane potential to increase the spontaneous release of acetylcholine (ACh), and it also inhibits nuclear factor kappa B (NFκB) through the NKA-associated Src signaling pathway (Yamazaki et al., 2007; Škubník et al., 2021a). Otherwise, they are closely related to Wnt/*β*-catenin signaling pathway and MEK1/2-ERK1/2 signaling pathway (Wang et al., 2018; Wong et al., 2018). Here we review the pharmacological activity of CTS and their potential clinical applications, aiming to provide a strong reference for CTS as new and effective lead compounds to develop new drugs for the treatment of various clinical diseases.

## 2 Chemical Structure and Structure-Function Relationship of CTS

**FIGURE 1:**
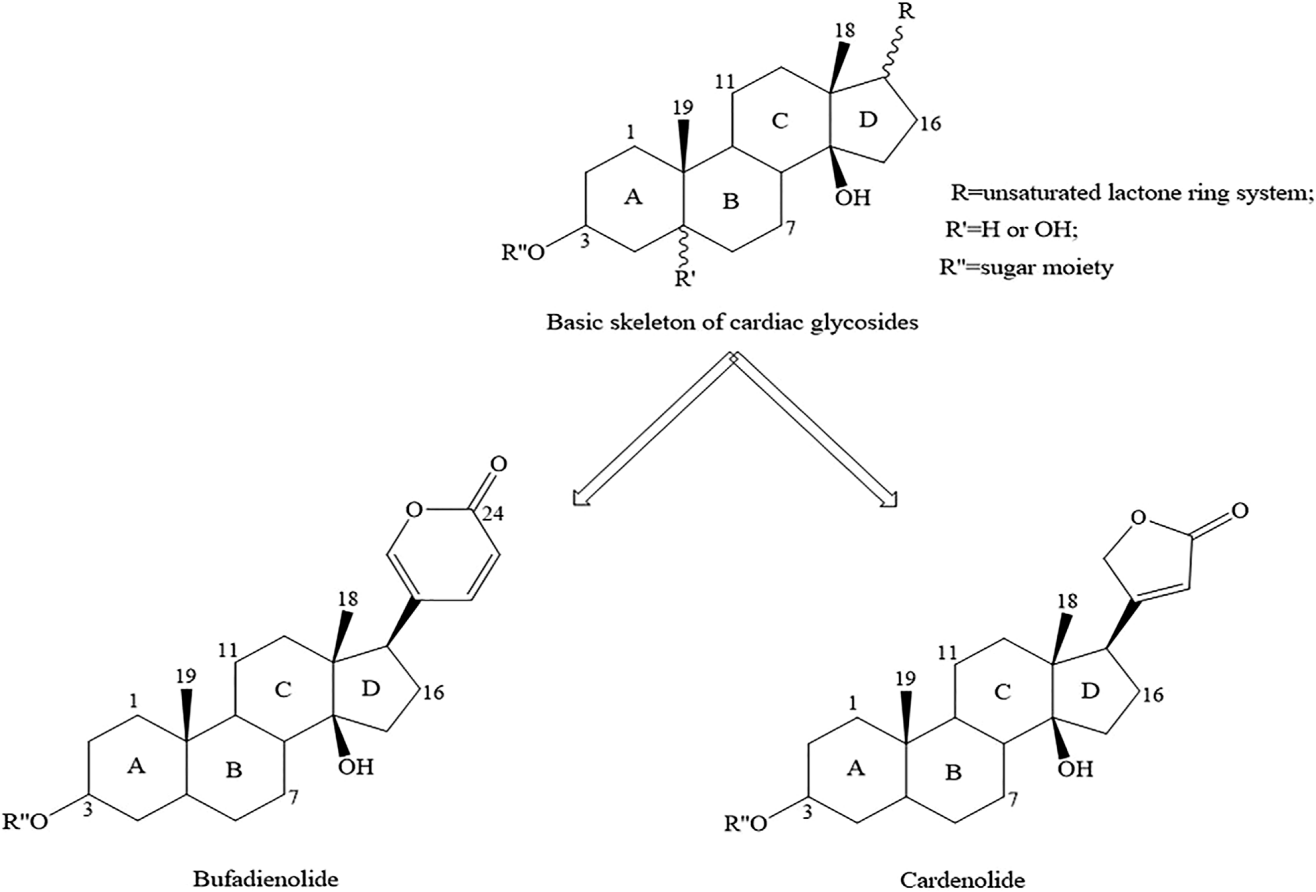
Basic skeleton of CGs. CGs is the most common type of CTS. The lactone moiety defines the functional class of each compound. Cardenolides contain a five-membered unsaturated butyrolactone ring, whereas bufadienolides contain a six-membered unsaturated pyrone ring.

CTS contains CGs and a fraction of non-glycosylated compounds including genines, bufalin, cinobufagenin and so on (Mijatovic et al., 2007). Since CGs occupy a vital position, the study of their structure is more conducive to clinical practice. The following description focus on the structure of CGs. The basic chemical structure of CGs is mostly composed of sugar residue, unsaturated lactone ring, and steroid nucleus. The unsaturated lactone ring determines the functional classification of each compound, which is divided into two categories according to the characteristics of its ring (Deng et al., 2020; Prassas and Diamandis, 2008) (Figure 1). One is bufadienolides, which contains a six-membered unsaturated lactone ring at C17, the other is cardenolides, which contains a five-membered unsaturated lactone ring at C17 (Deng et al., 2020). The latter binds to the target protein and exerts stronger biological activity than the former, and at the same time produces heavier toxicity (Chen, 2013).

CGs is the most common type of CTS. The lactone moiety defines the functional class of each compound. Cardenolides contain a five-membered unsaturated butyrolactone ring, whereas bufadienolides contain a six-membered unsaturated pyrone ring.

The chemical structure of CGs is closely related to their biological activities (Schönfeld et al., 1985; Kamano et al., 2002; Ye et al., 2004; Chen, 2013; El-Seedi et al., 2019). Sugar residues are attached to the 3-position *β*-OH of the nucleus, including *α*-hydroxysugars (glucose, 6-deoxysugar, and 6-deoxysugar-3-methyl, etc.) and *α*-deoxysugars (2, 6-dideoxysugar, 2,6-dideoxysugar-3-methyl, etc.). Sugar residues are related to the water solubility, antitumor activity, and pharmacokinetics (Ye et al., 2004; Chen, 2013; Ayogu and Odoh, 2020; Liu et al., 2020). In addition, the number of sugar residues also affects its biological activity, usually arranged like monoglycoside > diglycoside > triglycoside according to the pharmacological activity, which indicates that the main role is the first group of the nucleus (Chen, 2013; Ayogu and Odoh, 2020).

**FIGURE 2:**
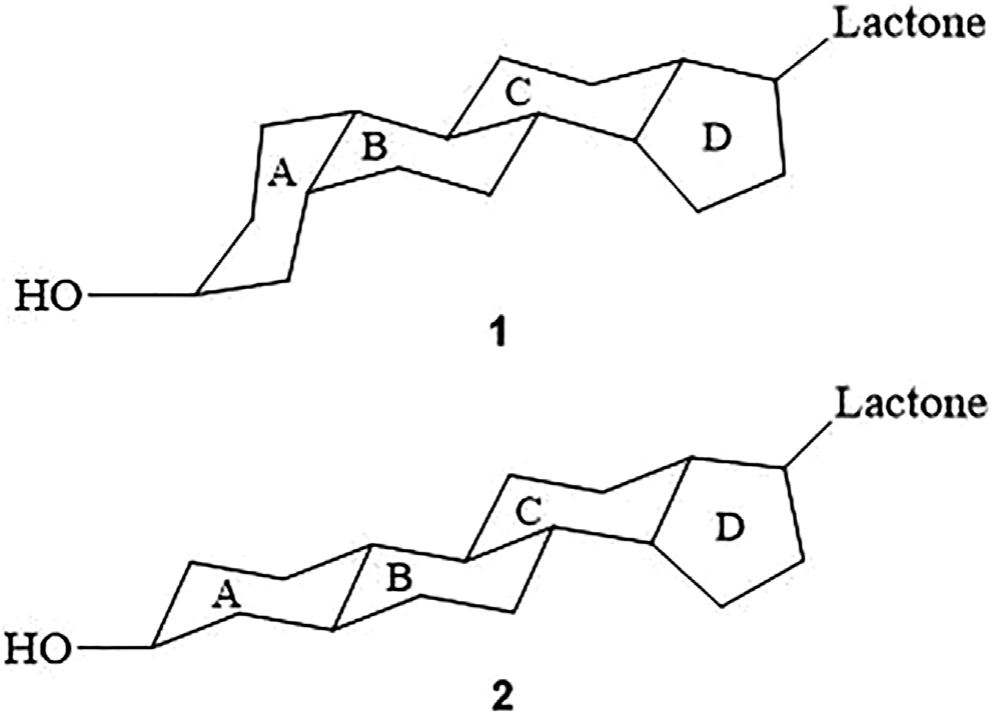
Spatial configuration of CTS. Ring A/B and ring C/D of compound 1 are cis-junction, while ring B/C is trans-junction; Ring A/B of compound 2 is transfused which is less common.

Aglycones are formed by combining a steroid nucleus and an unsaturated lactone ring. The steroid nucleus is the active component of CTS (Schönfeld et al., 1985). A/B and C/D cis-junction are the prerequisites for the effect of digitalis compounds (Schönfeld et al., 1985). Cis-junction of ring A/B is the majority modes in CTS (Chen, 2013) (Figure 2). Likewise, A/B cis-junction is the most effective for enhancing aglycone activity, such as Digitoxigenin (Schönfeld et al., 1985). A/B trans-junction is less, such as Uzarigenin, which is the most widely distributed in Asclepiadaceae (Malcolm, 1991).

Ring A/B and ring C/D of compound 1 are cis-junction, while ring B/C is trans-junction; Ring A/B of compound 2 is transfused which is less common.

## 3 Research Progress on the Pharmacological Activity of CTS

Cardiotonic activity of CTS in the cardiovascular system has been widely known, and even though the safe plasma concentration range is narrow, they still show irreplaceable scenarios in clinical practice (Malik et al., 2019; Pashazadeh-Panahi and Hasanzadeh, 2020). Furthermore, CTS have presented various activities in different tissues and systems, such as the broad anti-tumor effect, the broad antiviral effect, regulation of inflammatory factors, neuron protection and so on (Sibarov et al., 2012; Ayogu and Odoh, 2020; Škubník et al., 2021a; Škubník et al., 2021b).

### 3.1 Research Status of CTS in Cardiovascular Activity

Cardiovascular disease is the earliest application field of CTS, which is mainly used in the treatment of heart failure and arrhythmia.

#### 3.1.1 Study on the Role of CTS on Heart Failure

Cardiotonic steroid drugs are commonly used in the treatment of chronic heart failure (CHF). According to 2021 *Guidelines for Rational Drug Use at Primary Level in Chronic Heart Failure* published by the *Chinese Journal of General Practitioners*, the indication for digitalis drugs is patients with heart failure (HFrEF) who continue to have persistently decreased ejection fraction after using diuretics, angiotensin-converting enzyme inhibitors (ACEI)/angiotensin receptor blockers (ARB), *β*-blockers, and aldosterone receptor antagonists (Guidelines for rational drug use at primary level in chronic heart failure, 2021). Clinical studies have shown that digoxin can reduce the hospitalization rate of patients with HF but has no significant effect on all-cause mortality (Ziff et al., 2015; Malik et al., 2019). Lam PH et al. found that among hospitalized patients treated with *β*-blockers, concomitant use of digoxin reduced the rate of all-cause readmitted within 30 days but did not reduce mortality (Lam et al., 2018). This unique effect of digoxin persisted over a longer period of follow-up (4 years) (Lam et al., 2018). Recent studies have found that in the elderly population who discontinued pre-admission digoxin therapy can significantly affect the prognosis of patients, although they accepted guideline-directed medical therapies (GDMT), such as ACEI/ARB and *β*-blockers (Malik et al., 2019). The results of this study indicate that digoxin still plays an important role in the clinical treatment of CHF patients, and effectively improve cardiac function and prognosis.

The main mechanism of CTS in the treatment of CHF is to increase the concentration of Ca^2+^ in cardiomyocytes, and the related mechanisms are mainly as follow: CTS binding NKA secondary changes of sodium-calcium exchangers, and secondary calcium release from the sarcoplasmic reticulum. These mechanisms do not exist independently but are interrelated and mutually influential. NKA consists of two functional subunits, *α* and *β*. Four isotypes of *α* subunit (*α*_1_, *α*_2_, *α*_3_, *α*_4_) and four isotypes of *β* subunit (*β*_1_, *β*_2_, *β*_3_, *β*_4_) have been identified (Kaplan, 2002). Both *α* and *β* subunits are closely related to the functional activity of NKA, but each type of CTS binds and inhibits the *α* subunit of NKA (McDonough et al., 1990; Medina-Ortiz et al., 2021). The sodium-calcium exchanger (NCX) is a calcium transport system that controls intracellular calcium levels. There are two different modes of operation, namely, positive drive and reverse drive, which is usually positive drive. The reverse NCX is driven by Na^+^ influx, resulting in secondary Ca^2+^ elevation and Na^+^ discharge (Gerkau et al., 2018; Chovancova et al., 2020). NKA is inhibited by CTS, followed by changes in NCX activity. For example, NKA is inhibited by a low concentration of ouabain, and NCX positive driving force decreases, Ca^2+^ outflow slows down, and intracellular Ca^2+^ concentration increases relatively, which is mainly related to the specific blocking of NKA*α*_2_ subunit by ouabain (Swift et al., 2010). Changes in NKA activity, NCX reverse drive and other factors can cause Ca^2+^ transmembrane transport, thus triggering Ca^2+^ release from the sarcoplasmic reticulum (SR) (Bers, 2002). It should be noted that NCX reverse drive is not the main trigger factor (Reuter et al., 2005). Among various mechanisms that cause sarcoplasmic reticulum to release Ca^2+^, the main mechanism is the opening of the L-type Ca^2+^ channel triggers I_Ca-L_ formation (Bers, 2002; Reuter et al., 2005). And then entering intracellular Ca^2+^ binds to the ryanodine receptor (RyR) in the sarcoplasmic reticulum to Ca^2+^-induced Ca^2+^-release (CICR). After CTS inhibit NKA, intracellular Na^+^ accumulation triggers NCX activity, resulting in Ca^2+^ influx, and the formed I_Na/Ca_ causes SR to release Ca^2+^, or directly activates outward I_Na/Ca_ through membrane depolarization to cause SR to release Ca^2+^ (Bers, 2002). In the state of muscle relaxation, troponin prevents actin contraction, while Ca^2+^ binds to troponin and changes its conformation, ensuring that the binding site of myosin and actin is not covered (Škubník et al., 2021b). Therefore, CTS increase intracellular Ca^2+^ concentration through the mechanisms to increase myocardial contractility.

#### 3.1.2 Study on the Role of CTS on Arrhythmia

CTS can be used in the clinical treatment of arrhythmias, including atrial fibrillation (AF), atrial flutter (Afl), and paroxysmal supraventricular tachycardia. Atrial fibrillation is the most common clinical arrhythmia characterized by rapid, disordered atrial excitation and irregular ventricular rhythm. Atrial flutter is rare and usually associated with AF, occurring before AF or in isolation (Herzog et al., 2017). Digitalis compounds can convert patients with AF and Afl and maintain normal sinus rhythm (Jelliffe, 2014). It has been reported that CTS can effectively treat paroxysmal supraventricular tachycardia in combination with allapinin, an antiarrhythmic alkaloid (Kapustnick et al., 2021).

Digoxin has played an important role in controlling the heart rate of AF patients since the 1960s (Scalese and Salvatore, 2017). In reports on safety and application, Lopes RD et al. found that a basic dose of digoxin was not associated with increased risk of death, but patients with slightly higher serum digoxin concentration had a significantly increased risk of death compared with patients who did not use digoxin (Lopes et al., 2018). In addition, epidemiological studies have found that digoxin use is associated with increased all-cause mortality in AF patients with HF, and the risk of all-cause mortality in AF patients without HF is significantly higher than that in AF patients with HF (Chen et al., 2015). The reasons for the clinical effects of digoxin on all-cause mortality, serious adverse events, quality of life, HF, and stroke are still unclear (Sethi et al., 2018). The heart rate reduction effect of digoxin is stronger than that of placebo but less than that of *β*-blockers, while the long-term effects of digoxin are still unclear (Sethi et al., 2018). In recent years, Kotecha D et al. found no significant difference in the quality of life in patients with permanent AF and HF who received low-dose digoxin or bisoprolol treatment for 6 months (Kotecha et al., 2020). Therefore, more large-scale clinical case investigations with high quality, low bias, and low random error are needed to comprehensively evaluate the clinical efficacy of digoxin in AF patients and the effectiveness of combined therapy strategies. Nevertheless, digoxin is still one of the most prescribed drugs for AF in the world. For AF patients who cannot control heart rate with other drugs, digoxin can be considered (Ferrari et al., 2020). At the same time, it is necessary for doctors to fully evaluate the risks and effects according to the actual situation of different patients, adopt personalized plans, and check serum digoxin concentration regularly to ensure drug safety.

**FIGURE 3:**
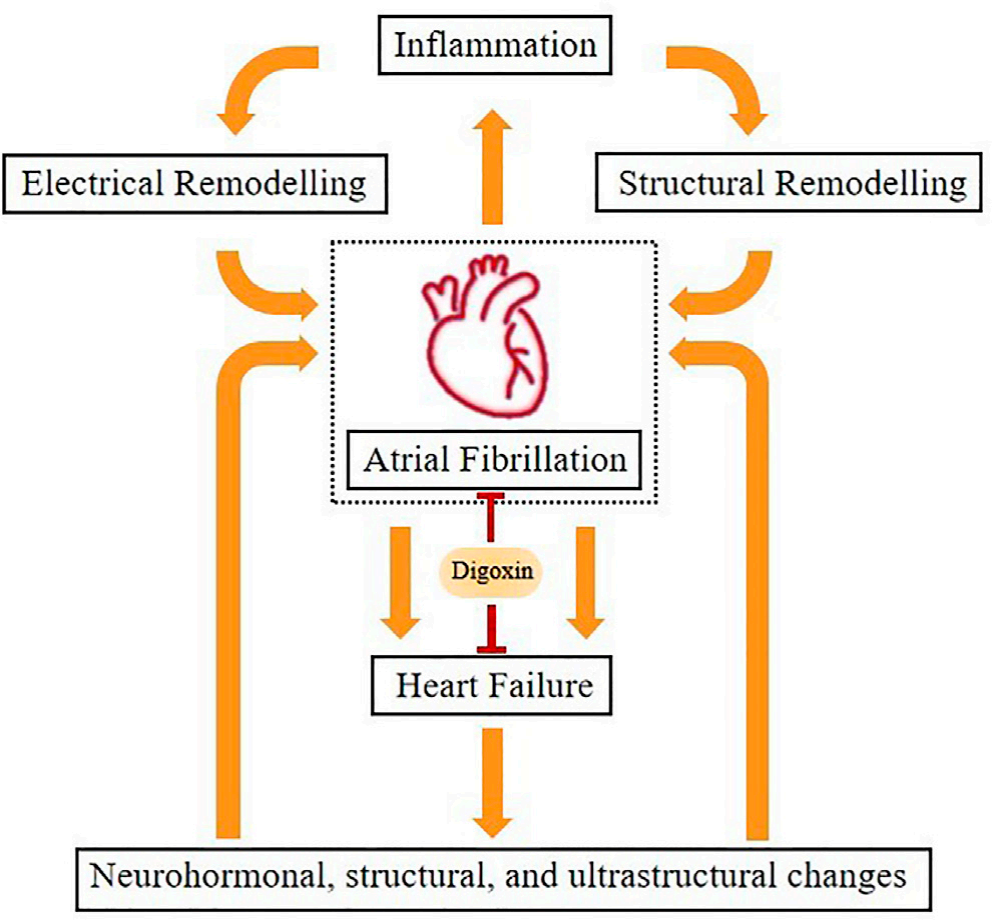
The relationship between heart failure and atrial fibrillation.

At present, rhythm control and rate control are two main strategies for the treatment of AF. Rhythm control aims to maintain sinus rhythm, while rate control aims to regulate the ventricular rate, thereby controlling symptoms and reducing the risk of complications. Digoxin mainly treats AF through the latter (Alobaida and Alrumayh, 2021). CTS enhance cardiac vagal nerve activity by promoting ACh release, which is related to the conversion of subthreshold stimulation to suprathreshold stimulation by CTS to sensitize vagal ganglia (Feinauer et al., 1986; Yamazaki et al., 2007). In addition, inflammation is not only the cause of the initial development of AF but also a factor for the continuation of arrhythmia (Boos et al., 2006). Chronic inflammation can induce atrial myoelectrical remodeling and tissue remodeling, which leads to the occurrence and development of AF. The pathophysiological process of AF can promote inflammatory response, leading to the phenomenon of AF begets AF (Hu et al., 2015). Changes in neurohormone, structure, and ultrastructure were observed in HF, which promoted the occurrence and development of AF, and AF also accelerated the development of HF (Carlisle et al., 2019) (Figure 3). Since CTS have a regulatory effect on inflammatory factors, it can be predicted that CTS may be involved in the inflammatory response process of various heart diseases (Škubník et al., 2021b).

#### 3.1.3 Study on the Effect of CTS on Vascular System

A potential reason for the beneficial effect of CTS in HF patients is that a slight vasodilation of resistance vessels followed by decreased diastolic blood pressure, which may be triggered by the neurohumoral mechanism caused by CTS (Kirch, 2001). Ouabain induces contraction of arterioles and veins in the forearm of normal subjects and, conversely, dilation of arterioles and veins in HF patients (Mason and Braunwald, 1964). In coronary artery studies, rapid injection of ouabain produced a short-acting systolic effect in subjects without HF (DeMots et al., 1976).

In addition, for CHF, CTS can enhance myocardial contractility to increase cardiac output, but it does not significantly increase cardiac output in normal subjects. This may be due to the direct contraction of CTS on vascular smooth muscles, which increases the peripheral resistance and counteracts its positive inotropic effect, limiting the increase of the cardiac output (Longhurst and Ross, 1985). Besides, CTS can reduce peripheral resistance and antagonize part of vasoconstriction through the decompression reflex mechanism (Longhurst and Ross, 1985). HF patients have high resting sympathetic tone, leading to vasoconstriction. CTS reduce sympathetic tone by improving cardiac output and peripheral blood flow (Kirch, 2001). The vasodilation effect caused by decreased sympathetic activity exceeds the direct contraction effect of CTS on vascular smooth muscles, resulting in decreased vascular resistance and increased cardiac discharge (Longhurst and Ross, 1985). It has been found that CTS have certain selectivity in the biological activities of healthy people and HF patients, which is expected to further develop the stretched clinical application of CTS.

What’s more, endogenous ouabain (EO) is a bridge between brain aldosterone and blood pressure and is involved in long-term blood pressure control (Leenen et al., 2020). Na^+^ and angiotensin II (ANG II) are two physiologically relevant stimulatory factors that increase the production and release of the hypothalamus EO, which their chronic central nervous system-mediated effects are mediated by (Leenen et al., 2020). EO reduces vascular NKA*α*_2_ activity and promotes cytosolic Ca^2+^ increase by activating NCX (Ferrari et al., 2006). It also enhances renal tubular Na^+^ reabsorption *via* activation of the renal NKA*α*_1_ induced by a Src-EGFr-dependent tyrosine phosphorylation pathway, which increases peripheral vascular resistances and blood pressure (Ferrari et al., 2006). Therefore, blocking ouabain antagonists could serve as a therapeutic target for novel antihypertensives (Ferrari et al., 2006). Rostafuroxin, a digitoxigenin derivative, has been regarded as an ouabain-inhibitor counteracting the ouabain blood pressure effects (Ferrari, 2010). For any given blood pressure level, rostafuroxin may be conferred an organ damage protection greater than some available drugs by selectively blocking a molecular mechanism common to both hypertension and organ damage (Citterio et al., 2021). Michél Strauss et al. found that marinobufagenin (MBG), one of the compounds of the venom of the Bufo marinus toad, can achieve antihypertensive effects by inhibiting renal NKA and promoting natriuresis, and inhibit NKA of vascular smooth muscle cells which has a vasoconstrictive effect (Michél Strauss et al., 2019).

Above all, we look forward to its more possible clinical applications and directions in cardiovascular diseases.

### 3.2 Research Status of CTS in Tumor Diseases

Cancer is the second leading cause of death in human diseases globally, and its morbidity and mortality are increasing year by year, which have evolved into critical problems worldwide (Mattiuzzi and Lippi, 2019). Unfortunately, resistance to chemotherapy is a common disadvantage of treating different types of cancer. Therefore, it is one of the Frontier goals of cancer research to find new chemotherapeutic drugs, either with feasible synthetic routes or from nature (Fu et al., 2019). Recent studies have shown that in addition to the treatment of HF and arrhythmia, CTS may also be used to treat tumor-related diseases (Ayogu and Odoh, 2020).

Prostate cancer (PCa) is the second leading cause of death in men (Mattiuzzi and Lippi, 2019). According to the estimates of the American Cancer Society, about 26% of new cancer cases in men are caused by PCa, and PCa can be as high as the second in expected cancer deaths, second only to lung cancer (Siegel et al., 2021). It has been reported that bufalin, digoxin, and digitalin can be used in the treatment of PCa (Ayogu and Odoh, 2020). Chang, Y. M.et al. found that ouabain inhibit human prostate DU 145 cancer cells *via* inducing cell morphological changes, reducing cell viability, promoting G0/G1 phase arrest, and inducing apoptosis and DNA damage (Chang et al., 2019). Wang, F. et al. also found that Proscillaridin A, a cardiotonic steroid which is clinically used to treat cardiovascular diseases. Proscillaridin A has the effect of slowing the progression of PCa through inhibiting PCa cell proliferation, migration, and invasion and inducing apoptosis in virto (Wang et al., 2020).

**FIGURE 4:**
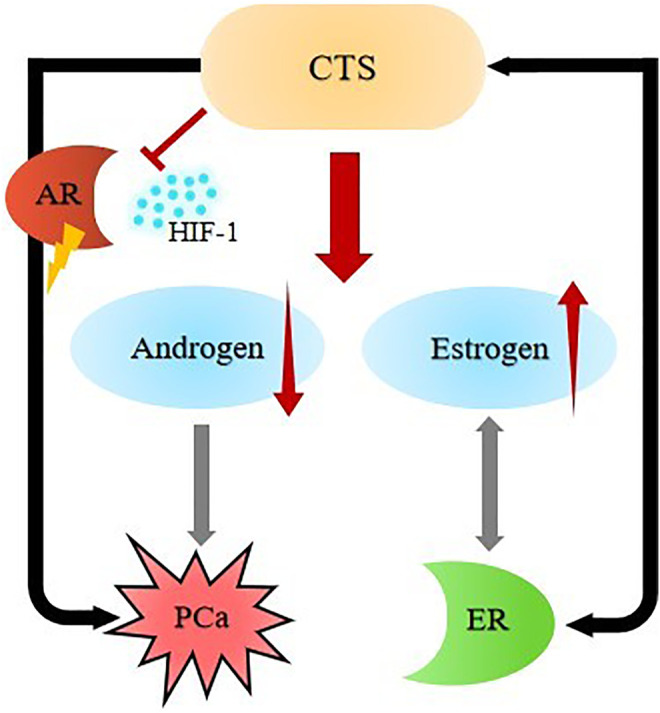
Biological activity of CTS in PCa.

In addition, other studies have found that long-term use of digoxin can increase serum estrogen level and decrease plasma testosterone level (Stoffer et al., 1973) (Figure 4), and testosterone level is linearly positively correlated with PCa growth (Yassin et al., 2019). Therefore, the effect of CTS on sex hormones may be one of the mechanisms of its preventive effect on PCa.

Endogenous and exogenous CTS affect the regulatory process of transcription factors by interacting with nuclear receptors (NR), such as the immune system, hormone management, body defense, cancer, etc. Nuclear receptors can be divided into multiple subfamilies. Estrogen receptor-like subfamily includes estrogen receptor (ER) and androgen receptor (AR) (Karaś et al., 2020). Due to the similar structure of CTS and estradiol (the most important estrogen), both can bind to ER (Biggar, 2012) (Figure 4). It is speculated that the prevention mechanism of CTS on PCa may also affect the regulation of transcription factors by binding to nuclear receptor ER. At the same time, it has also been found that CTS inhibit the growth of PCa cells by down-regulating hypoxia-inducible factor 1 (HIF-1) and blocking key genes of the AR-mediated signaling pathway (Lin et al., 2009; Li et al., 2012). In addition, there are also progesterone derivatives that can bind to digitalis receptors and some of them can be synthesized by CTS (LaBella et al., 1989; Templeton et al., 1997). It suggests that it is of great significance to have an insight into the therapeutic targets of diseases by regulating the binding process of compounds to cardiotonic steroid receptors in sex hormone-related diseases.

Nonetheless, Banerjee, M. et al. found that loss of NKA*α*_1_ is an important mechanism for the increased metastatic potential of epithelial–mesenchymal transition and PCa (Banerjee et al., 2021). The mechanism is based on increased activation of the NKA*α*_1_/Src signalosome complex and subsequent endocytosis in cancer cells, whereas ouabain and other CTS stimulate cell signaling by activating NKA*α*_1_/Src receptor complex which leads to endocytosis (Banerjee et al., 2021). Therefore, some CTS have certain tumorigenesis tendency, which could promote the production and development of PCa.

In some preclinical studies of PCa, CTS have been shown to have antitumor effects (Chang et al., 2019). But in another study, it has been found to be tumorigenic (Banerjee et al., 2021). Even an increased risk of PCa specific mortality has been reported associated with the use of digoxin (Zhao et al., 2021). It still needs more research to prove the promoting or inhibiting effect of CTS on PCa, but there is no doubt that CTS have potential research value, and some cardiotonic steroid extracts or derivatives could become effective supplements for the treatment of PCa.

Furthermore, CTS have significant pharmacological activity against other tumors. For example, Rasheduzzaman M et al. found that lanatoside C and digoxin induce apoptosis through tumor necrosis factor-related apoptosis-inducing ligand (TRAIL), sensitizing human hepatoma cell lines (Huh-7 and HepG2) (Rasheduzzaman et al., 2019). Meanwhile, CTS can dose-dependently increase ROS production in Huh-7 and HepG2 cells, thereby enhancing the cytotoxic effect of TRAIL. Yu Z et al. found that bufalin inhibits the development of hepatitis B virus-associated hepatocellular carcinoma through AR dephosphorylation and cell cyclin-related kinase degradation (Yu et al., 2020). Therefore, regulation of ROS generation and AR phosphorylation may be a new target for hepatoma therapy.

In addition, the antitumor mechanism of CTS is closely related to the Wnt/*β*-catenin signaling pathway, which may become an important regulatory pathway for its antitumor activity. For example, Wang J et al. found that bufalin can down-regulate ASCL2 gene expression by down-regulating the Wnt/*β*-catenin signaling pathway, thus preventing invasion and metastasis of gastric cancer cells (Wang et al., 2018). Zhao H et al. found that bufalin combined with cisplatin can overcome or delay drug resistance in the treatment of gastric cancer (Zhao et al., 2016). These results indicate that CTS have potential values in the treatment of gastric cancer.

What’s more, some clinical case studies have reported that CTS are associated with an increased risk of breast cancer and overall mortality (Osman et al., 2017; Zhang et al., 2017). However, other studies have confirmed that CTS play a significant inhibitory role in triple-negative breast cancer, which inhibits tumor proliferation by hampering the expression of the eIF4A1 subunit in the eukaryotic translation initiation factor (eIF) 4F complex. This complex may be a potential chemotherapy target for triple-negative breast cancer (Howard et al., 2020). Therefore, we speculated that the antitumor activity of CTS has a wide range of efficacy, which depends on the mechanism and type of tumor.

**FIGURE 5:**
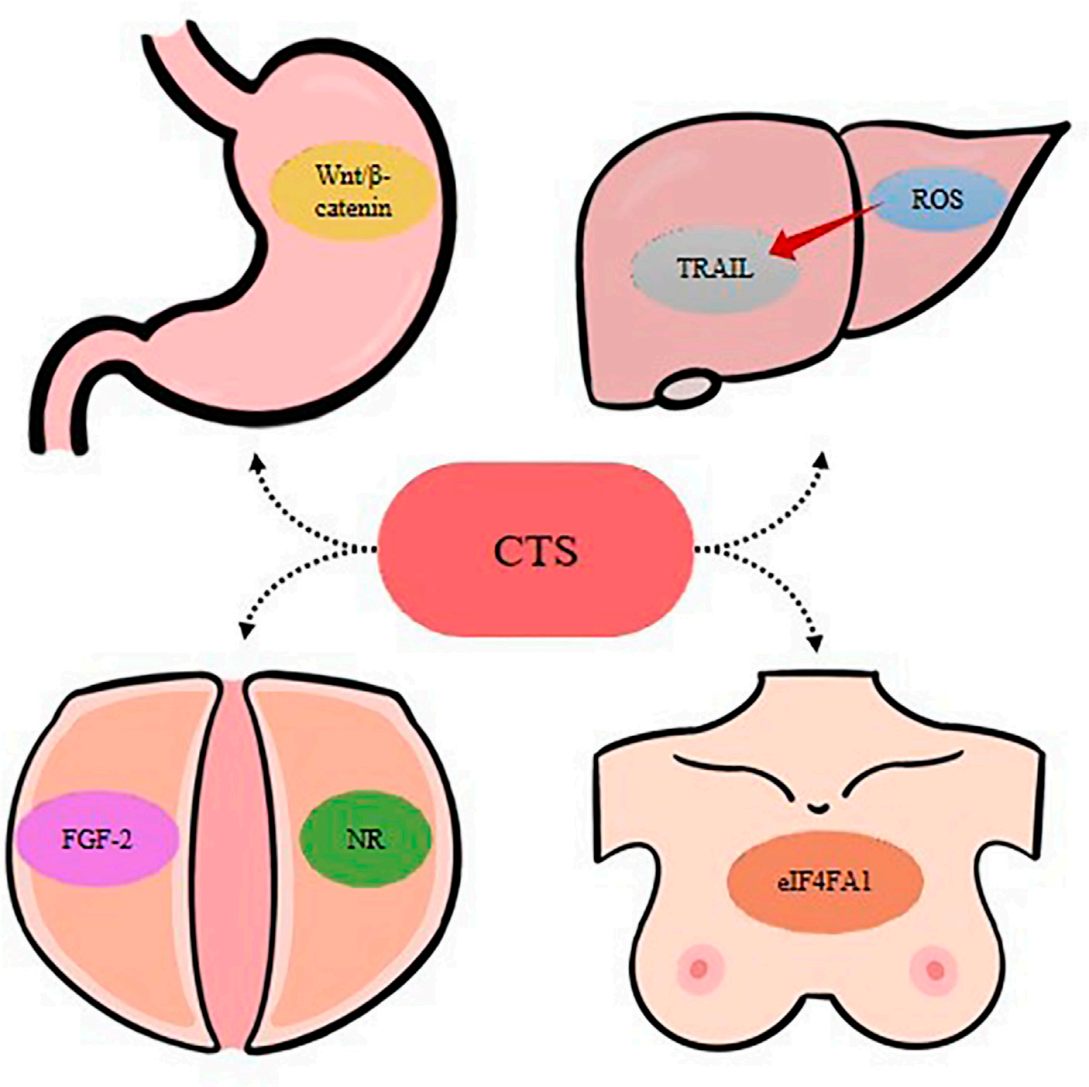
Schematic diagram of the antitumor mechanisms of CTS.

In summary, the antitumor pharmacological activity of CTS is mainly based on its role as a powerful immune stimulant compound, which effectively inhibits the growth of cancer cells and tumor development through the synergistic effect of the immune system (Figure 5). The potential antitumor activity of CTS is very valuable for clinical research, which opens a new idea for further development of antitumor drugs.

### 3.3 Effects of CTS on Viral Infectious Diseases

Although CTS drugs have not been approved for clinical treatment of viral infectious diseases, more and more evidence show that CTS drugs have extensive antiviral effects (Škubník et al., 2021a). CTS are effective against both DNA and RNA viruses and have the property of targeting host cell proteins, helping to reduce therapeutic resistance, making them a promising new strategy for antiviral infection (Amarelle and Lecuona, 2018).

#### 3.3.1 Research on the Role of CTS on Human Immunodeficiency Virus

Highly active antiretroviral therapy is the main treatment for human immunodeficiency virus-1 (HIV-1) infection, but it is not enough to rely on only one treatment, and related problems of drug resistance, toxicity, and cost still exist (Laird et al., 2014). Laird GM et al. found that CTS can effectively inhibit the expression of the HIV-1 gene, which depends on NKA in the human body, but has nothing to do with the increase of intracellular Ca^2+^ induced by CTS (Laird et al., 2014). Other studies have found that digoxin can effectively inhibit HIV-1 infection by inhibiting the expression of genes involved in T cell activation and cell metabolism, and the expression frequency of these genes and adjacent genes is higher in HIV-1 wild-type than capsid protein single point mutation (N74D) type (Zhyvoloup et al., 2017). In addition, Singh S et al. found that Anvirzel, a defined aqueous extract of Nerium oleander containing oleandrin, can inhibit the expression of HIV envelope surface protein GP120 which is closely related to HIV infection (François and Balzarini, 2011; Singh et al., 2013). Secondly, Wong RW et al. found that CTS inhibited the expression of the HIV-1 gene by activating the MEK1/2-ERK1/2 signaling pathway (Wong et al., 2018). This also showed that this inhibition was unrelated to the increase of intracellular Ca^2+^ by CTS. These studies indicate that the anti-HIV-1 effect of cardiotonic steroid drugs on the one hand reduces the infectivity of the virus by inhibiting the gene expression of the virus, and on the other hand, it is related to the activation of the immune system of the body, which also suggests that it may be a potential development prospect of new anti-HIV drugs in the future.

#### 3.3.2 The Role of CTS on Cytomegalovirus

CMV is a globally prevalent DNA herpesvirus (Davis et al., 2017). CMV has strong immunogenicity and produces a large number of CD4^+^ and CD8^+^T cells and high antibody titers during an individual’s lifetime, so it is a virus of lifelong infection (Hill, 2018). Generally, the immune response of the body can effectively inhibit viral infection, so infection cases generally only occur in immunodeficient individuals (Hill, 2018). Convallatoxin is a cardiotonic steroid extracted from *Lily of the Valley* (Morimoto et al., 2021). Gardner TJ et al. found that convallatoxin can inhibit CMV invasion and slow down the proliferation of viruses that have invaded cells (Gardner et al., 2015). Cohen T et al. further confirmed the anti-CMV effect of convallatoxin and proposed that this activity was achieved by inhibiting cell uptake of methionine and thereby reducing viral translation proteins (Cohen et al., 2016). Subsequently, Mukhopadhyay R et al. found that digitoxin inhibits CMV replication through a novel pathway, that is, by activating the NKAα_1_ subunit dependent adenosine-activated protein kinase (AMPK) and autophagy, thereby inhibiting human cytomegalovirus (HCMV) replication (Mukhopadhyay et al., 2018). These results suggest that the antiviral effect of CTS is closely related to NKA. In addition, clinical case reports have found that the combination of cardiotonic steroid drugs (digoxin, digitoxin, and ouabain) with ganciclovir (GCV) can enhance the antiviral effect (Cai et al., 2014). It is suggested that CTS may contribute a new strategy for antiviral combination therapy. In conclusion, the above-mentioned studies showed the antiviral effect of CTS on CMV, and provided an important reference for the development of new anti-CMV drugs or clinical combination therapy for CMV.

#### 3.3.3 Research on the Role of CTS on SARS-CoV-2

The previous studies have shown that CTS have potential antiviral activity, and their antiviral activity has once again attracted widespread attention as the world is experiencing the Corona Virus Disease 2019 (COVID-19) pandemic. COVID-19 is an epidemic disease caused by novel coronavirus (SARS-CoV-2) infection, with over 500 million confirmed cases and over 6 million deaths have been reported globally as of 1 May 2022 (WHO, 2022). The global COVID-19 pandemic is one of the most devastating infectious diseases in history in terms of numbers of infections and human mortality (Zhou et al., 2022). Therefore, it is urgent to explore effective anti-SARS-CoV-2 drugs to control the epidemic from both prevention and treatment. Recently, new drugs have been introduced into the treatment regimen for COVID-19 (Pourkarim et al., 2022). Meanwhile, the pharmacological activity of CTS against SARS-CoV-2 has been gradually explored. Plante KS et al. found that preventive treatment of Vero cells with oleandrin significantly reduced the progeny virus production (Plante et al., 2020). In addition, Cho J et al. found that the titer of progenies treated with digoxin and ouabain single dose significantly reduced (Cho et al., 2020). Some scholars have proposed different explanations for the anti-SARS-CoV-2 action of CTS. The initial infection of alveolar cells by the SARS-CoV-2 requires the Receptor Binding Domain (RBD) on the viral Spike (S) protein to bind to the domain of the ACE2 (angiotensin converting enzyme 2) receptor protein on the cell surface (Caohuy et al., 2021). Some CTS drugs competitively inhibit ACE2 binding to the RBD, thereby blocking viral penetration into human lung cells and this process may occur early in the entry process (Caohuy et al., 2021). The mechanisms of CTS against SARS-CoV-2 differ from each other, such as ouabain inhibiting endocytosis at the viral entry stage, digitoxin inhibiting cytokine storm and digoxin inhibiting RNA synthesis (Liana and Phanumartwiwath, 2022). The most likely mechanism of CTS against SARS-CoV-2 is the inhibition of NFκB through the NKA-associated Src signaling pathway (Škubník et al., 2021a). NFκB is activated after SARS-CoV-2 infection, and digoxin directly interferes with viral replication by inhibiting NFκB and inflammatory cytokines (Souza et al., 2021). The world is experiencing a huge wave of infection with the SARS-CoV-2 omicron variant (Murray, 2022). Therefore, the active exploration and development of antiviral activities of compounds such as CTS have promoted the development of new drugs against SARS-CoV-2. The broad multi-targeted antiviral activity of CTS makes the study more meaningful.

In addition, CTS also have inhibitory activity against a variety of viruses. For instance, Boff, L. et al. discovered two novel semi-synthetic cardenolides—C10 and C11, which can reduce the transcription of herpes simplex virus and impair the synthesis of proteins of herpes simplex virus (Boff et al., 2019; Boff et al., 2020). In addition, they also have inhibitory effects on against acyclovir-resistant herpes simplex virus type 1 strains, which makes them possible to be candidate drugs against herpes simplex virus (Boff et al., 2019; Boff et al., 2020). Moreover, Ebola virus, Chikungunya virus, dengue virus and other viruses are also in scope (Škubník et al., 2021a). The main antiviral mechanism of cardiotonic steroid drugs is to disrupt the early and late activities of the virus cycle by disrupting the ion balance, triggering host cell autophagy or various signal cascades, to obstruct the virus life cycle (Škubník et al., 2021a). This mechanism suggests that CTS may have broad-spectrum antiviral activity and have great potential for clinical antiviral drug development.

### 3.4 Pharmacological Activity of CTS in Nervous System

At present, more and more studies have found that some cardiotonic steroid drugs also show significant pharmacological activities in neurological diseases. For example, CTS are involved in psychophysiological processes such as regulation of mental symptoms and protection of nerve cells through the regulation of neurotransmitters and homocysteine (HCY) (Sibarov et al., 2012; Lopachev et al., 2019; Ivanova et al., 2020).

#### 3.4.1 The Role of CTS on Neurotransmitter-Related Diseases

Dopamine (DA) is a neurotransmitter synthesized in the central nervous system and peripheray. The signaling pathways triggered by the activation of dopamine receptors are involved in the occurrence and development of various diseases of the nervous system, such as Parkinson’s disease, schizophrenia, Huntington’s disease, attention deficit and hyperactivity disorder, and addiction (Klein et al., 2019). Singh SV et al. found that endogenous CTS levels in the prefrontal cortex of patients with bipolar disorder were lower than those of normal subjects (Singh et al., 2020). Other studies have found that intraventricular injection of ouabain can cause manic-like hyperactivity in rats (el-Mallakh et al., 1995). Valvassori, S. S. et al. used the rat model of intraventricular injection of ouabain again in the study of manic conversion experiments in bipolar disorder (Valvassori et al., 2022). They found ouabain induced a manic-like effect in rats which was evident on the 7th day and rats exhibited depression-like behavior at 14th days when the manic-like effect gradually subsided (Valvassori et al., 2022). Lopachev A et al. found that the effect of ouabain is caused by the activation of the D_2_ receptor, and blocking the D_2_ receptor can prevent ouabain-induced hyperactivity and rigid movement in mice (Lopachev et al., 2019). Yu HS et al. found that activation of tyrosine hydroxylase in the ERK1/2 signaling pathway plays an important role in the manic-like hyperactivity model induced by ouabain in rats (Yu et al., 2011). It is speculated that endogenous CTS are involved in the pathophysiology of bipolar disorder through various mechanisms, and the corresponding biological activity may be related to its concentration. In addition, high concentrations of ouabain can increase the effect of extracellular DA concentration by reducing the uptake of DA by cells (Lopachev et al., 2019). The mechanism could help treat depression, cognitive impairment, and Parkinson’s disease.

In addition, glutamate (Glu) is the central nervous excitatory neurotransmitter, which is released by neurons into the synaptic cleft and binds to ionic or metabolic receptors to transmit excitement message, and overactivation or inhibition of its receptors may produce cytotoxic reactions, and the excitoxicity of Glu induced is one of the many neurodegenerative diseases’ common signs (Olney, 1994; Iovino et al., 2020). In addition, the accumulation of large amounts of cytoplasmic Ca^2+^ has also been proposed to one of the most important triggers of the mechanism of cell death (usually apoptosis), and Glu induces Ca^2+^ homeostasis imbalance, which together contributes to neuronal damage (Khodorov, 2004; Giorgi et al., 2012). Sibarov DA et al. found in their study that in the process of excitoxicity damage to neurons, low-dose ouabain could accelerate the excretion of Ca^2+^ in neurons through NCX, improving cytoplasmic calcium overload and reducing apoptosis events (Sibarov et al., 2012). It is suggested that CTS can regulate Ca^2+^ homeostasis imbalance induced by glutamic acid and provide a new treatment for neurodegenerative diseases as a new neuroprotective agent.

#### 3.4.2 Study on the Effect of CTS on HCY

Hyperhomocysteinemia is considered as a risk factor for stroke and an independent risk factor for intracranial atherosclerotic stenosis (Liu et al., 2019; Moretti and Caruso, 2019). In addition, it also aggravates many neurodegenerative diseases, including Parkinson’s disease and Alzheimer’s disease (Miller, 2000; Huang et al., 2005; Hu et al., 2013). Therefore, the control of nerve damage caused by HCY is an important means to prevent the occurrence of cerebrovascular diseases and alleviate the symptoms of Parkinson’s disease and Alzheimer’s disease. HCY-induced neurodegeneration is caused by neuronal permanent plasma membrane depolarization and cytosolic Ca^2+^ overload of neurons, known as excitotoxic stress (Ivanova et al., 2020). Studies have found that ouabain can effectively antagonize neuronal damage caused by high HCY (Ivanova et al., 2020). Ouabain rapidly reduced the effects of calcium overload and reduced mitochondrial intima potential induced by Glu or HCY in neurons with short duration (4 h) excitotoxic injury. In neurons that undergo prolonged (24 h) excitotoxic injury, ouabain plays a neuroprotective role by triggering an intracellular neuroprotective cascade dependent on protein kinase A (PKA) and protein kinase C (PKC) to prevent neuronal apoptosis. Under excitotoxic stress, the maintenance of ion gradient on the plasma membrane by NKA consumes ATP and increases the burden of mitochondria, resulting in a decrease in mitochondrial intima voltage, while the long-term increase of intracellular Ca^2+^ concentration and mitochondrial dysfunction can lead to cell apoptosis through signal cascade (Ivanova et al., 2020). Ivanova MA et al. found that after co-treatment with HCY and ouabain, the accumulation intracellular Ca^2+^ and the decrease of mitochondrial intima potential were prevented to alleviate cellular ATP deficiency, thus reducing neuronal death events (Ivanova et al., 2020). This neuroprotective effect was closely related to Ca^2+^ clearance promoted by NCX (Ivanova et al., 2020). These studies suggest that low concentrations of ouabain protect nerve cells from excitotoxic damage by regulating the NKA/NCX system and reducing calcium overload in nerve cells.

In conclusion, ouabain has a lot of pharmacological activity and dose-dependent characteristics in the study of the nervous system, which not only participates in the regulation of nervous and mental activities but also has good clinical significance for central degenerative diseases and neuron injury.

### 3.5 Effects of CTS on Autoimmune and Inflammation-Related Diseases

CTS also have immune system activity and have potential application in the treatment of autoimmune and inflammatory diseases. At present, guidelines for indications and clinical use of CTS for the treatment of the diseases have not been licensed, but the pathogenesis and the development process of autoimmune diseases are complex and changeable, and now there is still a lack of specific medicine clinical treatment of these diseases, so it is particularly important to explore multi-target drugs regulating the immune system. It has been found that CTS can participate in the regulation of key immune cells in the pathological development of Rheumatoid arthritis (RA), atherosclerosis (AS), airway inflammation, and neuroinflammation (Shi et al., 2016; Zeitlin et al., 2017; Saeed et al., 2020; Jansson et al., 2021). Although there are rarely clinical applications of CTS in these diseases, it is rational to speculate that CTS will become an effective drug for immunotherapy in the future.

**FIGURE 6:**
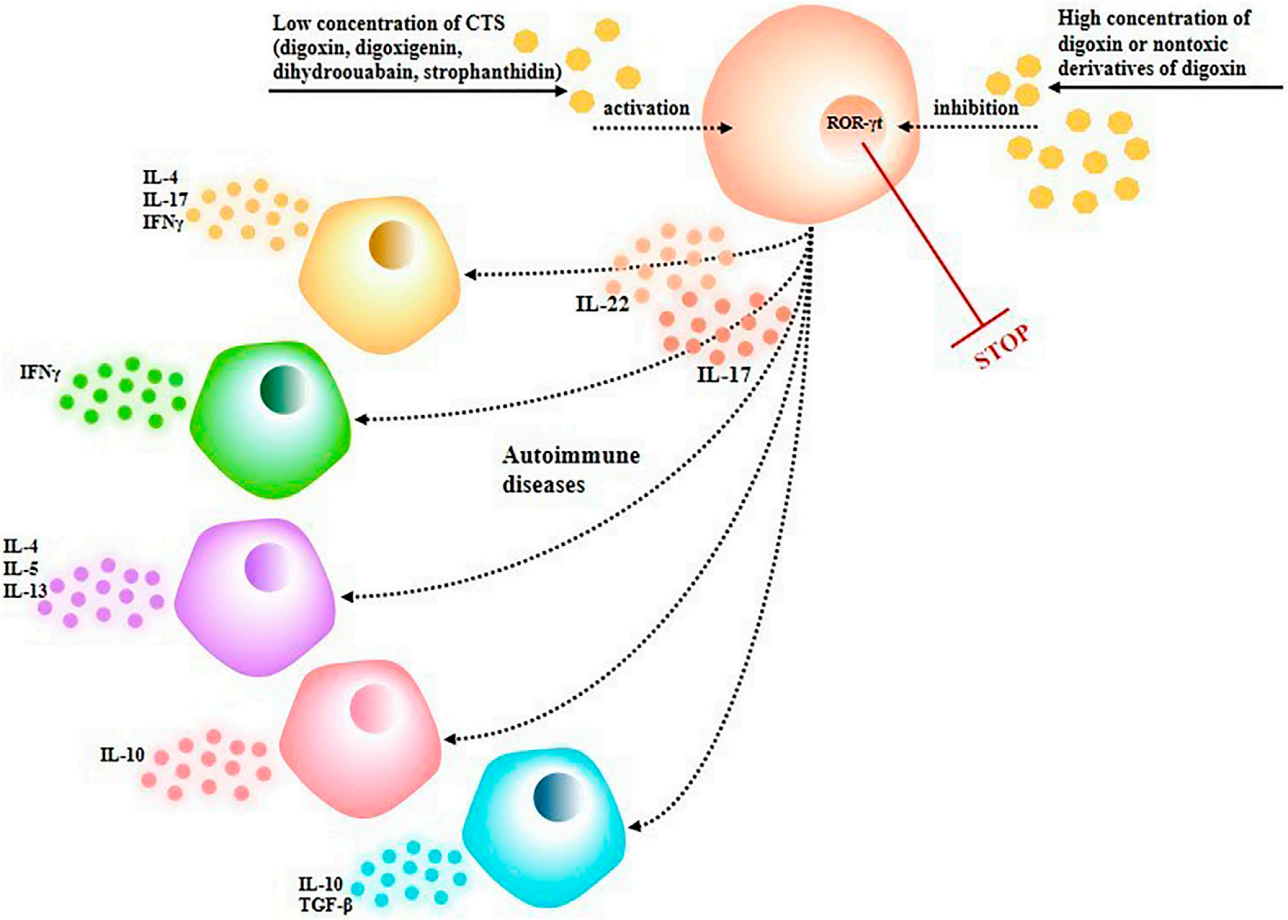
Effects of different concentrations of CTS on the cellular immune system.

RA is a systemic autoimmune disease characterized by synovial hyperplasia accompanied by persistent arthritis, and its pathogenesis is associated with complex factors, including smoking, obesity, pathogen infection, synovial injury, synovial fibroblast hyperplasia, etc. (Lin et al., 2020). Saeed H et al. found that digoxin is closely related to cytokine expression in peripheral blood mononuclear cells (PBMCs) of RA patients, and digoxin can significantly reduce cytokines such as IL-1*β*, IL-6, and IL-17, among which the effect was most prominent against IL-6 (Saeed et al., 2020). Second, digoxin inhibits Th17 cell differentiation by decreasing the expression of retinoic acid receptor-related orphan receptor *γ*T (ROR-*γ*T) and down-regulating the expression of IL-1*β*, IL-6, IL-17, and IL-23 in RA patients’ PBMCs (Saeed et al., 2020). Škubnik J et al. further studies found that the activity of CTS is concentration-dependent, that is, a low concentration of CTS can activate ROR-*γ*T, while a high concentration of CTS inhibited ROR-*γ*T transcription (Škubník et al., 2021b). ROR-*γ*T activation induces Th17 to produce IL-17 and IL-22, which further activates immune cells such as follicular B-helper T cells (TFH), type 1 and 2 helper T cells (Th1, Th2), type 1 regulatory T cells (TR1), and regulatory T cells (Treg) to promote autoimmune diseases (Škubník et al., 2021b) (Figure 6). The above-mentioned mechanism suggests that CTS have a multi-pathway regulatory effect on the inflammatory development process involving immune cells. In addition, although the existing clinical treatment of disease-improving antirheumatic drugs (DMARDs) can effectively improve the symptoms of the disease and prevent the progression of RA to a certain extent, the side effects and treatment cost limit the long-term and widespread use of patients (Lin et al., 2020). Therefore, the exploration of novel anti-inflammatory drugs with low toxicity, high efficiency, and low cost is one of the clinical objectives for the treatment of RA, and CTS can be used as a potential lead compound for the treatment of RA, which has broad application prospects.

AS is a common chronic inflammatory disease, which is an inflammatory injury process of arterial vascular endothelial and vascular wall involving a variety of inflammatory cells and inflammatory factors (Zhu et al., 2018). Extensive studies have found that the occurrence and development of AS can be effectively prevented and treated through anti-inflammatory and immune regulation. Shi H et al. found that lanatoside C can promote the occurrence of AS, which may be due to the up-regulation of SR-A and CD36 gene expression mediated by peroxisome proliferator-activated receptors (PPARs), resulting in increased oxLDL uptake and foam cell formation (Shi et al., 2016). Although this finding was obtained from animal studies, lanatoside C is a cardiotonic steroid approved by the US Food and Drug Administration (FDA) for the treatment of HF and arrhythmia (Shi et al., 2016). Therefore, it is widely used in clinical practice and needs attention.

In addition, Zeitlin PL et al. found that digoxin inhibited the transcription of inflammatory factors (IL-8 and IL-6) in patients with airway inflammation, and this effect could be detected after digoxin reached serum steady-state concentration (generally 4 weeks) (Zeitlin et al., 2017). Secondly, Galvao J et al. found that ouabain negatively regulates ovalbumin-induced allergic airway inflammation by inhibiting the production of IL-3 and IL-4 (Galvão et al., 2017). The foregoing indicates that the biological activity of CTS in inhibiting immune-inflammatory response can play an effective therapeutic role in respiratory immune inflammation-related diseases.

A variety of neurological diseases also have immune-inflammatory pathophysiological features, such as Alzheimer’s disease, Parkinson’s disease, Amyotrophic lateral sclerosis and multiple sclerosis, which promote disease occurrence through neuron injury and blood-brain barrier dysfunction (Ransohoff, 2016). Most trials for new therapeutic agents in AD are conducted in patients already receiving cholinesterase inhibitors, memantine, or both and are thus new types of add-on treatment. (Cummings et al., 2019). In addition, neuroinflammation contributes to the progression of neurodegenerative diseases, and if the efficacy of CTS in this area is confirmed, it could be a breakthrough in the treatment of such diseases. Fortunately, Jansson D et al. identified digoxin and lanatoside C as inflammatory modulators of blood-brain barrier cells by screening 1280 FDA-approved drugs (Jansson et al., 2021). Therefore, a cardiotonic steroid is expected to be used as a new drug in the treatment of neuroinflammatory-driven degenerative diseases.

In conclusion, current studies have found that CTS have an obvious inhibitory effect on the immune-inflammatory response, which has shown their prospects of development and application in the clinical treatment of immune-inflammatory diseases related to a variety of systems in the future. However, their mechanism of action is still very complex, which requires further in-depth study and confirmation.

## 4 Summary and Outlook

The pharmacological activity of CTS in cardiovascular diseases is the earliest research field discovered and applied in clinical practice. By inhibiting NKA, CTS eventually cause the increase of intracellular Ca^2+^ concentration to exert a positive inotropic effect and reduce heart rate by improving vagal nerve activity. With the continuous progress of research on the biological activity of CTS in the multidisciplinary field of medicine, CTS are gradually found to be a class of compounds with a variety of complex pharmacological activities, and are expected to develop novel therapeutic drugs in the treatment of clinical multidisciplinary diseases (respiratory system, tumor, diseases of the nervous system and immune inflammation-related diseases, etc.).

In tumor diseases, CTS have a significant antitumor effect on some tumors. The antitumor mechanism may be involved in inhibiting the metabolism of cancer cells and inhibiting the growth of cancer cells by increasing the ROS content in cancer cells, affecting the secretion of cytokines, interacting with nuclear receptors to affect gene expression, and regulating signal transduction pathways. The expression of the NKA subunit in some cancer cells may be altered compared with healthy tissues (Mijatovic et al., 2007). Studies have shown that the antitumor effect of CTS is mainly through the non-transport function of NKA, that is, the activation of signal transduction function (Bejček et al., 2021). The specific antitumor efficacy and mechanism of action need further study. Anti-tumor drugs are easy to produce drug resistance, so it is the goal of cancer research to find an anti-tumor drug that can be synthesized stably and has multiple subspecies. The diversity of sugar residue structure of the natural cardiotonic steroid drugs expands the variety and biological activity of the drug, and sugar residue also has a great impact on its anti-tumor activity and pharmacokinetic properties (Ye et al., 2004; Chen, 2013). Therefore, on the premise that the effective antitumor site substituents remain unchanged, the adverse reactions of CTS in antitumor activity can be avoided by further modification of sugar residues. In addition, the blood drug concentration is also a major factor affecting the effect of drugs, and it has been found that humans may not be able to tolerate the high concentration of CTS used in most preclinical studies (Osman et al., 2017). The goal of optimal clinical effect at minimum concentration can be achieved by altering the dynamics and tissue distribution of the drug in the body by changing the dosage form and preparation technology.

Secondly, the antiviral activity of CTS involves non-specific and highly specific effects. The non-specific mechanism is related to the blocking of NKA and regulation of the balance of intracellular and extracellular ions, and the specific mechanism is mainly the interaction with NKA*α*_1_subunit (Škubník et al., 2021a). The related mechanisms affect many viruses such as HIV, CMV, SARS-CoV-2 and so on. The broad antiviral activity exhibited by CTS is an uplifting discovery, although we still need to continue to explore more molecular mechanisms, especially the antiviral targets of CTS. However, CTS, one drug with multiple drug targets, can treat a variety of viral infectious diseases, which is a worthy research topic for both clinical and scientific research. Therefore, we expect that CTS will become a new broad-spectrum antiviral drug and play a significant clinical effect on the treatment of more viruses.

The pharmacological activity of CTS in the nervous system, on the one hand, protects neurons from the toxic damage of Glu or HCY by regulating the NKA/NCX system, thus inhibiting apoptosis cascade caused by intracellular ion imbalance (Ivanova et al., 2020). On the other hand, it provides a new idea for reducing the risk factors of cerebrovascular diseases and improving the symptoms of neurodegenerative diseases. In autoimmune inflammatory diseases of the nervous system, most treatments are still hormone-based, such as multiple sclerosis, neuromyelitis optica and acute myelitis. However, hormone therapy is a double-edged sword. Long-term use will cause adverse reactions such as femoral head necrosis, osteoporosis, and glucorticoid induced glaucoma. Therefore, the regulatory role of CTS in autoimmune inflammatory diseases of the nervous system is expected to become a new alternative or supplementary hormone therapy.

The pharmacological activity of CTS in immune inflammation-related diseases is related to the activity of regulating the functions of immune cells and cytokines (Škubník et al., 2021b). The effects of CTS in regulating inflammation in different systems are divergent. When using them clinically, attention should also be paid to the production of side effects, especially in patients with cardiovascular diseases complicated by immune inflammation-related diseases. As inflammatory diseases have always been a crucial and difficult topic, CTS have expanded the research direction for studying the targets of such diseases and could become an effective supplement to inflammatory regulators.

In summary, CTS not only exert their pharmacological effects in the cardiovascular system through the adjustment of NKA/NCX , but also exhibit abundant research results in other four main systems, including tumors, viral infections, nervous system diseases and immune inflammation-related diseases. In addition, we also look forward to the research progress of CTS in other fields. There are many benefits to develop new applications based on existing drugs such as shortening the development cycle, saving development costs, and owning safety verification. Well-known examples are the development process of aspirin and thalidomide. The research progress of CTS in multi-system diseases have expanded the direction of their clinical research and CTS could be used as a lead compound to be promoted in the drug treatment of multi-system or multi-disease in the future.

## Glossary

ACEI: angiotensin-converting enzyme inhibitors
ACh: acetylcholine
AF: Atrial fibrillation
Afl: Atrial flutter
AMPK: adenosine-activated protein kinase
AR: androgen receptor
ARB: angiotensin receptor blockers; HF: heart failure
AS: atherosclerosis
CMV: Cytomegalovirus
CTS: cardiotonic steroids
CGscardiac glycosidesCHF: cardiac glycosidesCHFchronic heart failure
CICR: Ca2+-induced Ca2+-release
COVID-19: Corona Virus Disease 2019
DA: Dopamine
DMARDs: disease-improving antirheumatic drugs
EDLF: endogenous digitalis-like factor
eIF: eukaryotic translation initiation factor
EO: endogenous ouabain
ER: estrogen receptor
FDA: Food and Drug Administration.
FGF-2: fibroblast growth factor-2
GCV: ganciclovir
GDMT: guideline-directed medical therapies
Glu: glutamate
HCMV: human cytomegalovirus
RBD: Receptor Binding Domain
HCY: homocysteine
HIF-1: hypoxia-inducible factor 1
HIV-1: human immunodeficiency virus-1
IC50: 50% inhibitory concentration
MBG: marinobufagenin
NCX: sodium-calcium exchanger
NFκB: nuclear factor kappa B
NKA: sodium and potassium pump
NR: nuclear receptors
PCa: prostate cancer
RA: Rheumatoid arthritis
ROR-γT: retinoic acid receptor-associated orphan receptor γT
ROS: reactive oxygen species
RyR: ryanodine receptor
PBMCs: peripheral blood mononuclear cells
PKA: protein kinase A
PKC: protein kinase C
PPARs: peroxisome proliferator-activated receptors
SR: sarcoplasmic reticulum
TFH: follicular B-helper T cells
TR1: type 1 regulatory T cells
TRAIL: tumor necrosis factor-related apoptosis-inducing ligand
Treg: regulatory T cells
